# Evaluation of Ozonated Water’s Efficacy in Rinsing Equipment in a Processed Fishery Products Industry

**DOI:** 10.3390/foods15213954

**Published:** 2026-11-04

**Authors:** Chiara Di Vittori, Gianluigi Ferri, Maria Laura Fidani, Matteo Grieco, Annarita Festino, Alberto Vergara

**Affiliations:** 1 Department of Veterinary Medicine, Post-Graduate Specialization School in Food Inspection “G. Tiecco”, University of Teramo, SP 18, 64100 Teramo, TE, Italy; gferri@unite.it (G.F.);; 2 Foods Import F.lli Monti SPA, via Gabbiano 18, 64013 Corropoli, TE, Italy

**Keywords:** ozone, food industry, eco-friendly sanitation, cod

## Abstract

Ozonated water may represent a complementary approach to improve routine hygiene during fish processing. This study evaluated its effectiveness in reducing microbial contamination on food-contact surfaces of a cod-processing machine during scheduled production breaks and at the end of production. Tap water and ozonated water were compared under routine industrial conditions by monitoring Total Mesophilic Counts (TMC) and Total Psychrotrophic Counts (TPC) at multiple time points throughout the working day. Both treatments reduced microbial loads, but ozonated water achieved significantly greater reductions in TPC during morning and end-of-day rinsing and in TMC during morning rinsing after Holm’s correction for multiple comparisons. No significant differences were observed during lunch rinsing or for TMC during end-of-day rinsing. Microbial loads increased again during subsequent processing periods under both treatments. After final chlorine-based sanitation, neither TMC nor TPC differed significantly between treatments after Holm’s correction. Overall, ozonated water improved the effectiveness of selected rinsing procedures under real processing conditions and should be considered complementary to conventional end-of-day cleaning and disinfection. Its use during production breaks may therefore represent a practical complementary measure for controlling microbial accumulation in fish processing environments.

## 1. Introduction

Fishery products are considered highly perishable foods. In fish processing facilities, food-contact surfaces are continuously exposed to raw fish, tissue residues, process water and handling activities, creating multiple opportunities for microbial transfer and accumulation on equipment [1]. Recent investigations in commercial rainbow trout processing have identified conveyor belts, evisceration and filleting equipment as sites of relevant microbial contamination and have provided evidence of potential transmission of microorganisms from processing equipment to fish tissues [2].

Maintaining the microbiological hygiene of food-contact surfaces is therefore essential in seafood processing operations. Seafood processing environments are particularly challenging from a hygienic perspective, as high humidity, variable temperatures and the presence of organic residues can favour microbial persistence [3]. Contamination can occur continuously throughout the working day as equipment and conveyor surfaces are repeatedly exposed to fish material, processing residues and handling activities. As a result, microbial loads may progressively increase between daily sanitation procedures, highlighting the need for interventions that can control contamination during ongoing production.

In industrial practice, complete cleaning and disinfection procedures are generally performed at the end of the working day, whereas simpler rinsing interventions may be carried out during scheduled production breaks to remove residual fish material and limit the accumulation of contamination before processing resumes. Water rinsing represents a simple and operationally compatible intervention; however, its antimicrobial effect is associated with the physical removal of microorganisms and organic residues rather than with direct microbial inactivation. The identification of approaches capable of enhancing the microbiological effectiveness of routine rinsing procedures could therefore provide an additional tool for maintaining hygienic conditions during production without interrupting normal processing activities.

Ozone has attracted considerable interest as a potential complementary technology for microbial control in the food industry [4]. Owing to its strong oxidising capacity, ozone can inactivate a broad range of microorganisms, while its rapid decomposition to oxygen means that it does not leave persistent chemical residues on treated surfaces. Ozone has been investigated for microbial control in different food-processing applications, including the treatment of equipment and other food-contact surfaces, in both aqueous and gaseous forms [5,6].

The application of ozonated water may be particularly relevant for intermediate hygiene interventions because it combines the physical rinsing action of water with the antimicrobial activity of dissolved ozone. However, ozone efficacy is strongly affected by application conditions, including ozone concentration, contact conditions, surface characteristics and, particularly, the presence of organic matter [7]. Organic residues can rapidly consume dissolved ozone and reduce the amount available for microbial inactivation [8,9]. Studies conducted on seafood-processing surfaces have consequently reported variable efficacy depending on the type and condition of the treated surface [8].

Despite the increasing interest in ozone-based technologies, evidence concerning their application under routine industrial conditions remains limited. In particular, few studies have evaluated the use of ozonated water specifically as an intermediate rinsing treatment during the working day in fish-processing facilities, where surfaces are exposed to continuous product contact and residual organic material. Moreover, the effectiveness of such an approach needs to be assessed in comparison with the water-rinsing procedures already implemented under standard industrial operating conditions rather than only under controlled laboratory conditions.

The present study therefore aimed to evaluate the effectiveness of ozonated water in reducing microbial contamination on food-contact surfaces of a cod-processing machine during routine production breaks and at the end of production. Its effectiveness was compared with that of tap water under routine industrial operating conditions by monitoring Total Mesophilic Counts (TMC) and Total Psychrotrophic Counts (TPC) at multiple time points throughout the working day.

## 2. Materials and Methods

The study was conducted between 2024 and 2025 in a cod processing facility. The target equipment was a Target Batcher portioning machine (JBT Marel, Chicago, IL, USA) used to produce fixed-weight portions (400–550 g) of rehydrated cod prior to packaging.

Under standard operating conditions, the machine is rinsed with high-pressure cold water during the scheduled production breaks (mid-morning and lunch breaks) to remove product residues from food-contact surfaces. At the end of production, following the rinsing treatment and the corresponding surface sampling, Topaz^®^ CL4 (Ecolab S.r.l., Vimercate, Italy), an alkaline chlorine-based foaming detergent, at a working concentration of 5%, was progressively applied across the machine. The manufacturer’s recommended action time of approximately 15 min was operationally counted from the start of foam application to the start of the final tap-water rinse. The final rinse lasted approximately 5 min, and surface samples were collected immediately afterwards, with no intervening production.

The routine cleaning schedule adopted by the company is reported in Table 1.

### 2.1. Sample Collection

This study compared the effectiveness of ozonated water with that of tap water, used according to the company’s standard operating procedure, for rinsing the Target Batcher portioning machine during production breaks and at the end of the working day.

The trial was conducted over a 12-month period and included 24 sampling days on the same Target Batcher machine. Each month, tap-water rinsing was evaluated first, followed by ozonated-water rinsing in a different week, with at least one intervening week without experimental trials. This yielded 12 sampling days per treatment. Sampling took place on Mondays or Wednesdays, depending on the production schedule for the product under investigation and access to the processing facility. Sampling days were selected according to operational availability rather than through a formal randomization procedure.

Ozonated water was generated by a portable ozone system (Dino Purification Co., Ltd., Guangzhou, China) with a nominal ozone concentration of 2.0 ppm and a specified water flow rate of up to 10 L/min, and was applied through a hand-held spray wand [10]. Dissolved ozone concentration was not independently measured at the spray-wand outlet or on the treated surfaces during the trial. Both treatments used the same refrigerated mains water supply, supplied through the facility’s chilled-water tank, and were applied using the same spray wands for 10 min per rinsing event to cover the entire machine. During ozonated-water application, water temperature and flow rate were monitored using the system’s display. Displayed water temperatures ranged from 2 to 3 °C, and flow rates were typically 9–10 L/min. The processing-room temperature displayed by the facility’s monitoring system was 7 °C.

During each sampling day, surface samples were collected at eight predefined time points: at the start of production, immediately before and after rinsing during the mid-morning break, immediately before and after rinsing during the lunch break, immediately before and after the end-of-production rinse, and after completion of the daily sanitation procedure.

**Figure 1:**
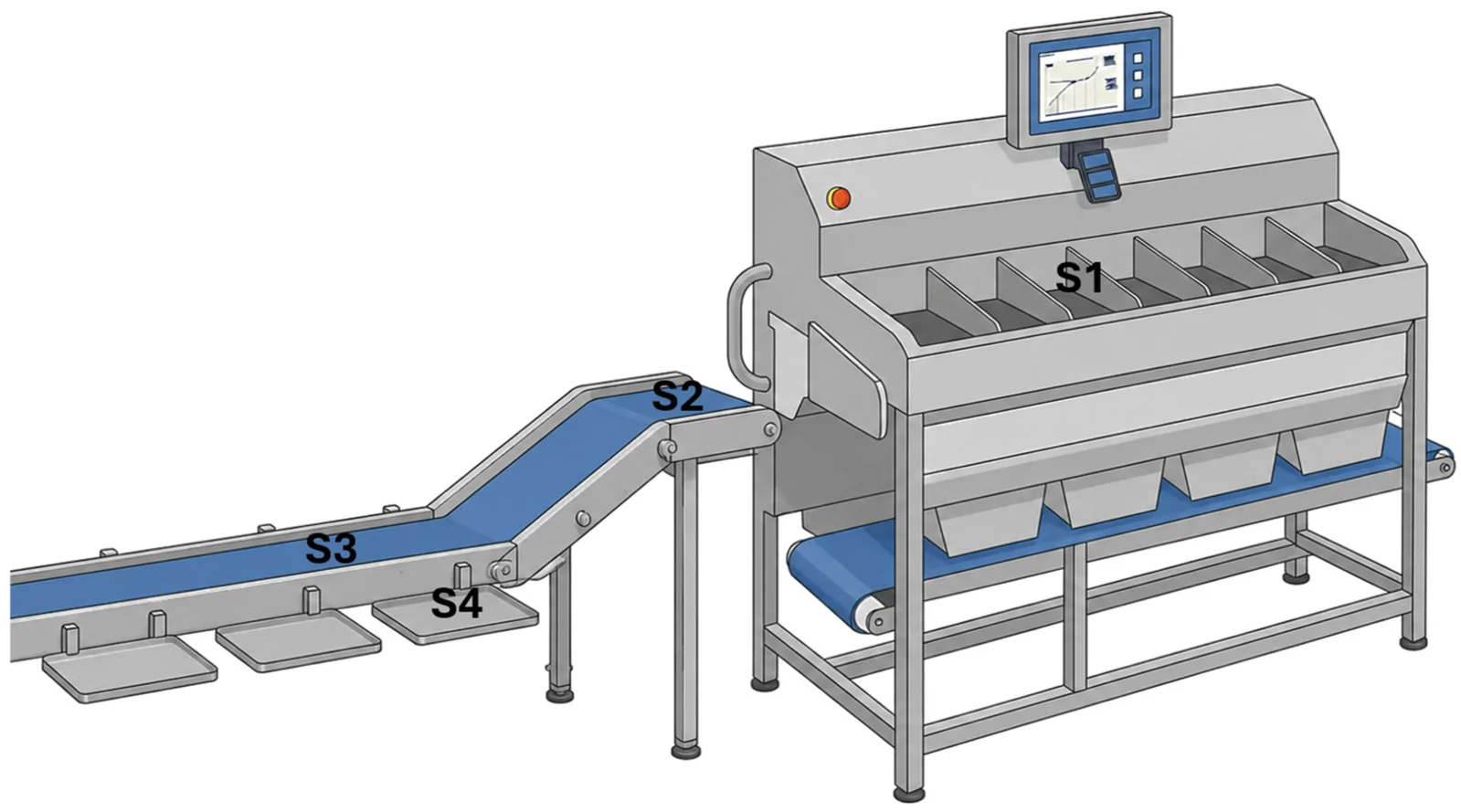
Schematic representation of the Target Batcher portioning machine and the selected sampling sites. Three sampling areas (S1–S3) were located along the conveyor belt, while one sampling area (S4) was located on a stainless-steel removable support shelf used by operators during production.

**Figure 2:**
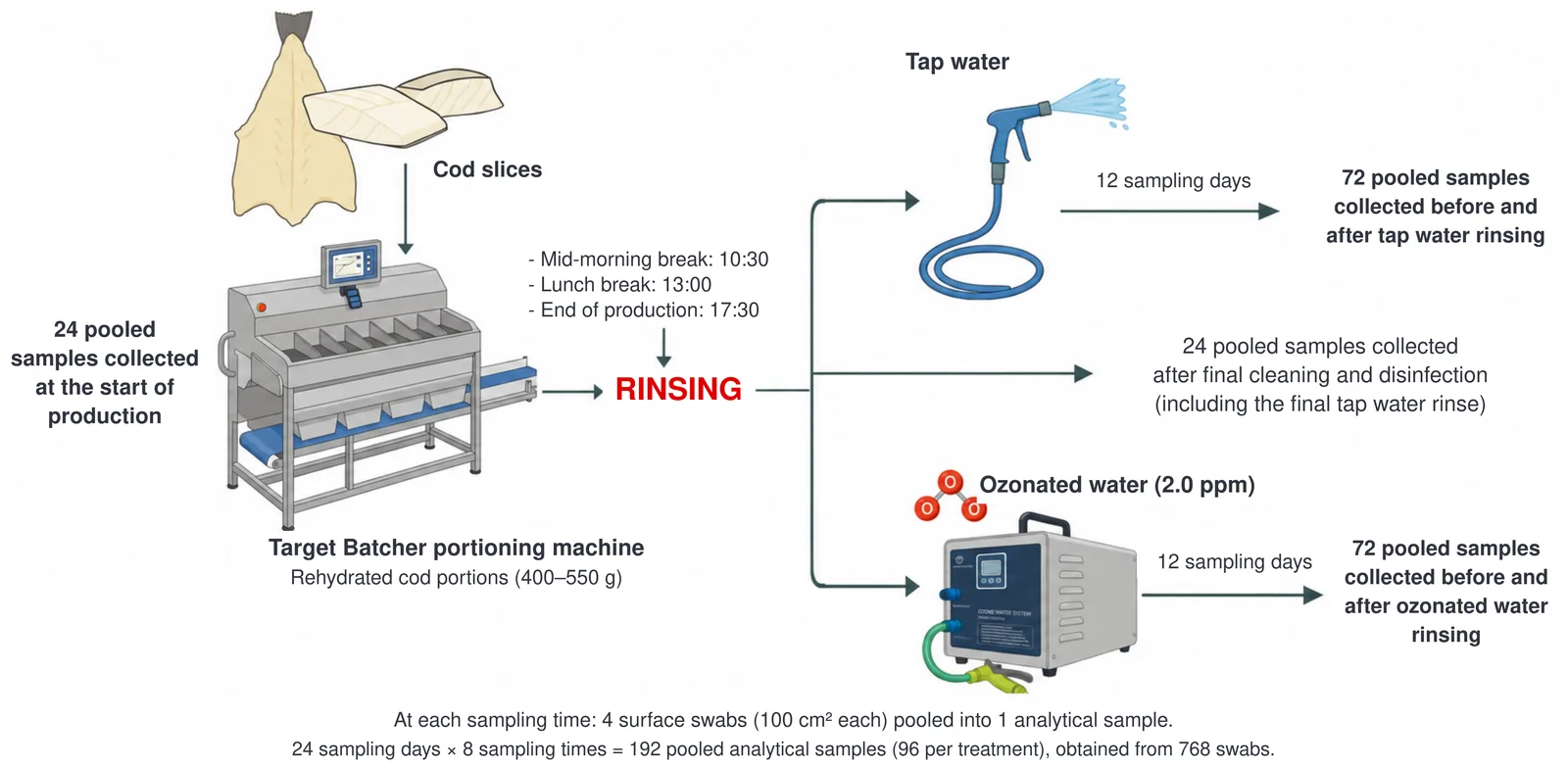
Sampling design for tap water and ozonated water rinsing treatments. At each sampling time, four surface swabs were pooled into one analytical sample. The ozone concentration shown (2.0 ppm) is nominal and was not independently measured at the point of application. Arrows indicate the workflow and sampling stages.

Surface sampling was performed using the swab technique in accordance with UNI EN ISO 18593:2018 [11]. Four sampling areas (100 cm^2^ each) were delimited using sterile 10 × 10 cm templates and sampled from selected food-contact surfaces of the Target Batcher machine as shown in Figure 1. At each sampling time, a new sterile template was positioned within each predefined sampling zone. Sampling locations varied within these zones, and the same fixed 100 cm^2^ areas were not deliberately resampled. Three sampling areas were located on the conveyor belt, representing different sections of the product transport path, while the fourth area was located on the stainless-steel worktable used by operators during handling operations. At each sampling time, the four swabs were pooled into a sterile 15 mL Falcon tube containing 10 mL of Maximum Recovery Diluent (MRD, Oxoid Ltd., Basingstoke, Hampshire, UK). No neutralising agent was added to the sampling diluent. Overall, 768 surface swabs were collected and pooled in groups of four, yielding 192 analytical samples: 96 for the tap water treatment, and 96 for the ozonated water treatment. The sampling design is summarised in Figure 2.

### 2.2. Microbiological Analysis

Microbiological analyses included the enumeration of total mesophilic counts (TMC) and total psychrotrophic counts (TPC), performed according to ISO 4833-1:2013 [12] and ISO 17410:2019 [13], respectively.

Prior to analysis, the Falcon tubes containing the pooled swab samples in 10 mL of MRD were homogenised by vortex mixing. Serial ten-fold dilutions were then prepared in MRD. Dilutions up to 10^−3^ were analysed for samples collected at the start of production and from dirty surfaces prior to rinsing, whereas dilutions up to 10^−2^ were prepared for samples collected after rinsing with tap water or ozonated water and following the daily sanitation procedure.

For both microbiological parameters, 1 mL aliquots of the undiluted suspension and the prepared dilutions were plated in Plate Count Agar (PCA; Thermo Scientific™ Oxoid Standard Plate Count Agar; Oxoid Ltd., Basingstoke, UK) using the pour plate technique. TMC plates were incubated at 30 °C for 72 h, whereas TPC plates were incubated at 6.5 °C for 10 days according to the respective standard.

Counts were converted to CFU/cm^2^ by multiplying the colony count on the selected plate by the reciprocal dilution factor and the total recovery volume (10 mL), and dividing by the plated volume (1 mL) and the total sampled area (400 cm^2^). One colony on the plate inoculated with the undiluted suspension corresponded to a theoretical single-colony detection threshold of 0.025 CFU/cm^2^. Samples yielding no colonies at any dilution were reported as <0.025 CFU/cm^2^. For statistical analysis, these samples were assigned 0.025 CFU/cm^2^ before log_10_ transformation, corresponding to −1.60206 log CFU/cm^2^. All positive counts were log_10_-transformed without substitution.

### 2.3. Statistical Analysis

Microbial counts were expressed as log CFU/cm^2^ and analysed using JASP software (version 0.96.0.0; JASP Team, University of Amsterdam, Amsterdam, The Netherlands). Data are presented as mean ± standard deviation (SD).

To evaluate the cleaning efficacy of water and ozonated water, microbial reductions (Δ log CFU/cm^2^) were calculated for each cleaning event as the difference between pre-cleaning and post-cleaning microbial counts. Reductions were calculated separately for Total Mesophilic Counts (TMC) and Total Psychrotrophic Counts (TPC) at the morning, lunch, and end-of-day cleaning procedures.

Differences between treatments were assessed using independent-samples Student’s t-tests. Prior to analysis, normality of data distribution was evaluated using the Shapiro–Wilk test, while homogeneity of variances was assessed using Levene’s test. Cohen’s d was calculated as a measure of effect size. For comparisons showing departures from normality, Mann–Whitney U tests were additionally performed as sensitivity analyses.

To investigate microbial recontamination during production, the increase in microbial counts between post-cleaning and the subsequent pre-cleaning sampling point was calculated. Recontamination dynamics were analysed using independent-samples Student’s t-tests following the same assumptions and procedures described above.

Changes in microbial contamination throughout the working day were assessed separately for TMC and TPC using repeated-measures analysis of variance (RM-ANOVA), with sampling time as the within-subject factor and treatment (water or ozonated water) as the between-subject factor. Each sampling day represented a repeated-measures unit, with successive observations collected within that day (12 sampling days per treatment). The four swabs pooled at each sampling time constituted a single analytical sample and were not treated as independent replicates. The analysis included the start-of-production measurement and the six sampling times immediately before and after the three rinsing procedures. Counts obtained after the final chlorine-based sanitation were analysed separately using independent-samples Student’s t-tests. When the assumption of sphericity was violated, as assessed by Mauchly’s test, Greenhouse–Geisser corrections were applied. Partial eta-squared (ηp^2^) values were calculated to estimate effect sizes for ANOVA models.

Holm’s correction was applied separately to three families of primary treatment comparisons: six comparisons of microbial reductions across the three rinsing events and two microbial indicators; four comparisons of microbial increases across the two production intervals and two indicators; and two comparisons following final chlorine-based sanitation. Adjusted *p*-values are reported for these comparisons. Mann–Whitney U tests were used as sensitivity analyses, and their *p*-values are reported without adjustment.

For all analyses, statistical significance was set at *p* < 0.05.

## 3. Results

### 3.1. Cleaning Efficacy of Water and Ozonated Water

The effectiveness of water and ozonated water cleaning treatments was assessed by comparing the log reductions (Δ log CFU/cm^2^) of Total Mesophilic Counts (TMC) and Total Psychrotrophic Counts (TPC) at the morning, lunch, and end-of-day cleaning procedures.

For TMC, the mean reduction observed with ozonated water was significantly greater than that obtained with water during the morning rinsing procedure (1.93 ± 1.03 vs. 0.76 ± 1.01 log CFU/cm^2^, adjusted *p* = 0.040, Cohen’s d = 1.15). Although the mean reduction was also greater with ozonated water during the end-of-day cleaning procedure, the difference was not statistically significant after Holm’s correction (2.21 ± 1.22 vs. 1.20 ± 0.83 log CFU/cm^2^, adjusted *p* = 0.081, Cohen’s d = 0.97). No significant difference was detected during the lunch cleaning procedure (1.54 ± 1.22 vs. 1.02 ± 0.68 log CFU/cm^2^, adjusted *p* = 0.209).

For TPC, significantly greater reductions were observed with ozonated water during the morning cleaning procedure (2.17 ± 1.10 vs. 0.66 ± 0.91 log CFU/cm^2^, adjusted *p* = 0.008, Cohen’s d = 1.49) and the end-of-day cleaning procedure (2.14 ± 1.17 vs. 0.75 ± 0.98 log CFU/cm^2^, adjusted *p* = 0.023, Cohen’s d = 1.29). No significant difference was detected during the lunch cleaning procedure (2.12 ± 0.87 vs. 1.36 ± 1.11 log CFU/cm^2^, adjusted *p* = 0.149). The greater TPC reduction observed with ozonated water during end-of-day rinsing was also supported by the Mann–Whitney U test (U = 25.00, unadjusted *p* = 0.006).

**Figure 3:**
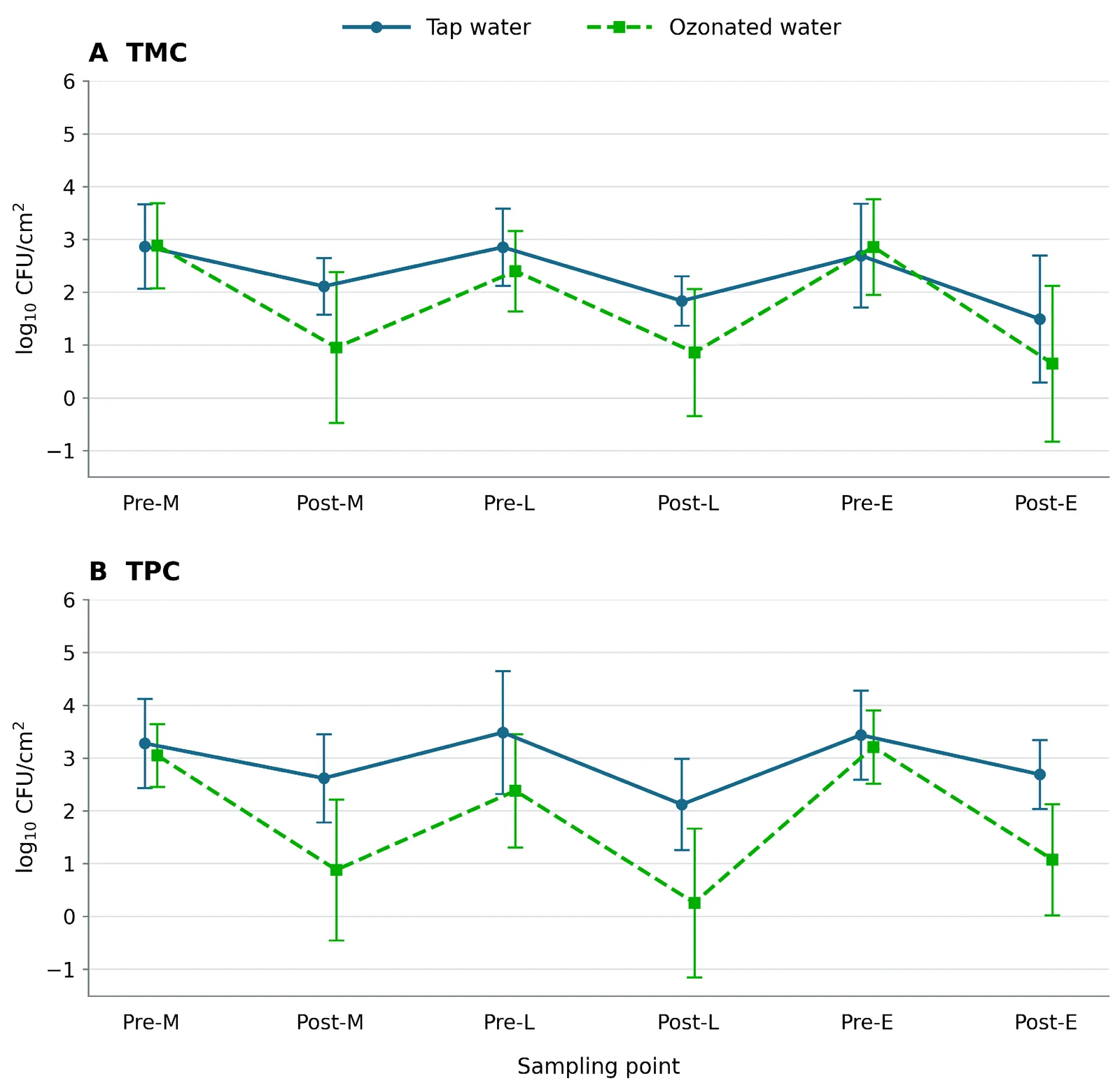
Temporal evolution of (**A**) Total Mesophilic Counts (TMC) and (**B**) Total Psychrotrophic Counts (TPC) on the Target Batcher machine during the working day under tap water and ozonated water cleaning procedures. Points represent mean log_10_ CFU/cm^2^, and error bars indicate ±1 standard deviation. Each mean represents 12 sampling days per treatment. Pre-M = before morning cleaning; Post-M = after morning cleaning; Pre-L = before lunch cleaning; Post-L = after lunch cleaning; Pre-E = before end-of-day cleaning; Post-E = after end-of-day cleaning.

The changes in microbial counts throughout the working day under tap water and ozonated water treatments are illustrated in Figure 3.

### 3.2. Recontamination Dynamics During Production

To investigate microbial recontamination during production, the increase in microbial load between post-cleaning and the subsequent pre-cleaning sampling point was calculated for both TMC and TPC.

For TMC, the mean increase between post-morning cleaning and pre-lunch sampling was 0.74 ± 1.02 log CFU/cm^2^ for the water treatment and 1.45 ± 1.21 log CFU/cm^2^ for the ozonated water treatment. The difference between treatments was not statistically significant (adjusted *p* = 0.273). Similarly, no significant difference was observed for TPC, with mean increases of 0.87 ± 0.86 and 1.50 ± 1.26 log CFU/cm^2^ for water and ozonated water, respectively (adjusted *p* = 0.273). The Mann–Whitney U test likewise detected no significant difference between treatments in the TPC increase between post-morning rinsing and pre-lunch sampling (U = 48.00, unadjusted *p* = 0.175).

During the second production period, the mean TMC increase between post-lunch cleaning and pre-end-of-day sampling was 2.00 ± 1.31 log CFU/cm^2^ for ozonated water and 0.86 ± 0.83 log CFU/cm^2^ for water. The difference did not reach statistical significance after Holm’s correction (adjusted *p* = 0.054, Cohen’s d = 1.04). For TPC, the increase was significantly greater with ozonated water (2.95 ± 1.79 vs. 1.31 ± 0.90 log CFU/cm^2^, adjusted *p* = 0.039, Cohen’s d = 1.16).

### 3.3. Temporal Evolution of Microbial Counts Throughout the Working Day

A repeated-measures ANOVA was performed to evaluate the effects of treatment (water vs. ozonated water) and sampling time on Total Mesophilic Counts and Total Psychrotrophic Counts throughout the working day.

For TMC, Mauchly’s test indicated a violation of the assumption of sphericity (W = 0.141, *p* = 0.008); therefore, Greenhouse–Geisser correction (ε = 0.597) was applied.

A significant effect of time was observed (F(3.585, 78.868) = 18.81, *p* < 0.001, ηp^2^ = 0.461). No significant main effect of treatment was detected (F(1,22) = 1.68, *p* = 0.208). A significant Time × Treatment interaction was observed (F(3.585, 78.868) = 3.52, *p* = 0.014, ηp^2^ = 0.138).

For TPC, Mauchly’s test indicated a violation of the assumption of sphericity (W = 0.086, *p* < 0.001); therefore, Greenhouse–Geisser correction (ε = 0.532) was applied. A significant effect of time was observed (F(3.193, 70.253) = 20.53, *p* < 0.001, ηp^2^ = 0.483). A significant main effect of treatment was also detected (F(1,22) = 11.18, *p* = 0.003, ηp^2^ = 0.337). In addition, a significant Time × Treatment interaction was found (F(3.193, 70.253) = 5.00, *p* = 0.003, ηp^2^ = 0.185).

Descriptive statistics for TMC and TPC at each sampling point throughout the working day are presented in Table 2.

### 3.4. Residual Differences After Final Chlorine Sanitation

To evaluate whether residual differences between treatments persisted after the final chlorine sanitation procedure, microbial counts measured after chlorine application were compared between water and ozonated water treatments.

For TMC, microbial counts were numerically higher in the ozonated water treatment, with mean values of 1.80 ± 0.81 log CFU/cm^2^ for water and 2.37 ± 0.42 log CFU/cm^2^ for ozonated water (t(22) = −2.124, adjusted *p* = 0.090, Cohen’s d = −0.867). The difference was not statistically significant after Holm’s correction.

For TPC, mean values after chlorine sanitation were 2.17 ± 0.82 log CFU/cm^2^ for water and 1.76 ± 1.17 log CFU/cm^2^ for ozonated water. No significant difference was detected between treatments (t(22) = 1.008, adjusted *p* = 0.324, Cohen’s d = 0.412).

## 4. Discussion

Food-contact surfaces in fish processing facilities are continuously exposed to microorganisms originating from raw fish, organic residues, processing aerosols and operators’ handling activities. Consequently, microbial loads on equipment surfaces fluctuate throughout the production day according to the balance between contamination events and routine cleaning interventions.

The present study showed a cyclic pattern of surface contamination under both cleaning protocols. Scheduled rinsing procedures performed during production breaks consistently reduced TMC and TPC, whereas microbial loads increased again during the subsequent processing period. This behaviour suggests that intermediate rinsing effectively limits microbial accumulation, although surface recontamination rapidly occurs once processing resumes. This dynamic has also been observed in commercial fish processing facilities, where conveyor belts, gutting machines and filleting equipment have been identified as critical contamination points because of their continuous exposure to fish tissues and processing residues [2]. Likewise, Miettinen et al. reported that, among 28 fish-processing factories sampled after cleaning and before production began, only two had total aerobic heterotrophic bacterial counts ≤5 CFU/cm^2^ in more than 90% of the surface samples [14]. This finding highlights the difficulty of achieving satisfactory surface hygiene even after routine cleaning. More recently, Reiche et al. reported an average reduction of 1.1 log CFU/cm^2^ in total aerobic counts following cleaning and disinfection in a salmon-processing plant [15].

Replacing tap water with ozonated water resulted in significantly greater reductions in TPC during the morning and end-of-day rinsing procedures and in TMC during morning rinsing. The mean TMC reduction was also greater with ozonated water at the end of production, but the difference was not statistically significant after Holm’s correction. These findings support the potential of ozonated water to improve selected rinsing operations under the industrial conditions studied, with the clearest statistical evidence observed for TPC. However, no significant advantage was observed during the lunch rinsing procedure for either microbial indicator. The antimicrobial activity of ozone is attributed to its strong oxidising capacity, which causes irreversible damage to bacterial cell envelopes and intracellular components. However, its efficacy is highly dependent on operational conditions, particularly the presence of organic matter, which rapidly consumes dissolved ozone and limits its availability for microbial inactivation [7,16]. Consequently, ozone efficacy decreases as the amount of organic residues on treated surfaces increases [8]. In our previous investigation conducted in the same cod-processing facility, ozonated water achieved substantially greater microbial reductions after the removal of visible organic residues than when applied immediately after processing on heavily soiled soaking boxes [10]. Beyond the effect of organic residues, the characteristics of the treated surface may also influence ozone efficacy. Crapo et al. [17] reported considerably greater microbial reductions on stainless steel than on polyethylene cutting boards contaminated with fish residues, indicating that both surface characteristics and the presence of residual fish material can affect treatment efficacy. Under routine industrial conditions, residual organic matter may therefore attenuate the antimicrobial activity of ozonated water. However, surface organic loads were not quantified in the present study, and samples from conveyor-belt and stainless-steel surfaces were pooled. The separate contributions of organic contamination and surface characteristics to treatment performance could therefore not be determined.

From an operational perspective, these findings are consistent with previous research investigating the practical application of ozonated water in seafood processing plants. Sopher et al. [18] suggested that the greatest benefit of ozonated water may be obtained when it is incorporated into routine equipment rinsing during production rather than applied after terminal sanitation procedures.

The main operational finding was the additional reduction in TPC achieved with ozonated water during morning and end-of-production rinsing. Mean TPC reductions were 2.17 and 2.14 log CFU/cm^2^ with ozonated water, compared with 0.66 and 0.75 log CFU/cm^2^ with tap water, during morning and end-of-production rinsing, respectively. The between-treatment differences remained significant after adjustment for multiple comparisons. Comparison with the facility’s routine tap-water rinse provides a practical benchmark for assessing the additional benefit of the ozonated-water system. Monitoring naturally occurring surface contamination also extends the evaluation beyond laboratory challenges with selected microorganisms. These observations, obtained over repeated sampling days in an operating facility, support the potential value of ozonated water for selected production-break rinses. The subsequent increases in surface counts also show why immediate rinsing performance and contamination during resumed production should be considered separately when assessing its practical contribution.

The larger increase in TPC between post-lunch and pre-end-of-production sampling with ozonated water should be interpreted alongside the lower post-rinse counts (0.26 vs. 2.12 log CFU/cm^2^ with tap water). Before end-of-production rinsing, mean TPC was numerically lower with ozonated water than with tap water (3.21 vs. 3.44 log CFU/cm^2^). Thus, the larger increase reflects the change from a lower starting count and does not establish faster microbial growth. These changes represent net microbial accumulation during production, potentially including microbial transfer from products and processing activities.

The TPC findings are particularly relevant to refrigerated fish processing because they describe the response of the culturable microbial population capable of growth at low temperatures. Studies with selected microorganisms have shown that susceptibility to ozonated-water spraying can differ between species [7]. The present study complements this evidence by evaluating naturally occurring surface contamination during routine production.

From an industrial perspective, ozonated water offers several practical advantages for routine hygiene management in fish processing plants. Unlike conventional chemical sanitisers, ozone rapidly decomposes into oxygen, a characteristic that supports its potential use during scheduled production breaks. In addition, ozone can be generated on site [19], which may facilitate its integration into routine production-break rinsing procedures.

Despite these advantages, ozonated water should not be regarded as a replacement for conventional end-of-day sanitation procedures. In the present study, chlorine-based sanitation remained part of the facility’s routine hygiene programme. Detectable TMC and TPC remained after this procedure; these results describe the residual culturable contamination and do not, by themselves, establish that an acceptable level of surface hygiene was achieved. Following final chlorine-based sanitation, mean TMC was numerically higher on days when ozonated water had been used, whereas mean TPC was lower. Neither comparison was statistically significant after Holm’s correction. On ozonated-water sampling days, mean TMC increased from 0.65 log CFU/cm^2^ after end-of-production rinsing to 2.37 log CFU/cm^2^ after final sanitation, approximately 20 min later, with no intervening production. These measurements describe counts recovered at two successive sampling points; they do not establish that sanitation caused the numerical increase. The study was not designed to determine the mechanism underlying this observation.

Chemical interactions between ozone and free chlorine are possible when the two oxidants coexist, as ozone reacts with hypochlorite and contributes to oxidant consumption [20]. Organic residues may also reduce the efficacy of chlorine-based disinfection, as demonstrated on food-contact surfaces under experimentally soiled conditions [21]. These published mechanisms provide general context but cannot explain the present findings without measurements of residual ozone, available chlorine and surface organic loads. The numerical differences observed here therefore do not demonstrate interference with subsequent chlorine-based sanitation.

The choice not to use ozonated water as a final rinse following the daily sanitation procedure was based on previous observations indicating that ozone may remove residual chlorine from chlorine-based detergents and sanitisers, potentially reducing their residual antimicrobial effect. Sopher et al. [18] demonstrated that washing cutting boards with ozonated water after the application of chlorine-containing cleaning agents resulted in increased microbial loads, probably due to the removal of residual chlorine combined with the introduction of moisture favourable for microbial survival and growth. Conversely, the authors suggested that ozonated water may be particularly advantageous when applied during scheduled production breaks, where its lack of residual activity is not detrimental and processing can rapidly resume.

The industrial setting provided practical relevance while allowing less control over production-related variability. The study involved a single machine in one processing facility, which limits generalisation to other equipment and settings. Pooling swabs from conveyor-belt and stainless-steel surfaces provided an overall assessment of the sampled food-contact surfaces but prevented comparisons between these surface types. Both treatments were evaluated each month on the same machine, but sampling days and treatment order were not randomized, leaving potential effects of production batches and other between-day differences. The absence of point-of-application ozone measurements and quantitative organic-load assessments limits mechanistic interpretation and transferability to other operating conditions. TMC and TPC were selected to assess overall culturable surface contamination; identification of the recovered microorganisms would provide complementary information on taxon-specific responses. Further studies combining these measurements across additional equipment and facilities would help define the conditions under which the observed benefit is most reproducible.

Overall, ozonated water should be viewed as a complementary technology within integrated hygiene management programmes, with its greatest potential lying in strengthening routine operational hygiene during production while preserving the role of conventional end-of-day cleaning and disinfection procedures.

## 5. Conclusions

In conclusion, ozonated water produced significantly greater TPC reductions than tap water during morning and end-of-day rinsing and significantly greater TMC reductions during morning rinsing on food-contact surfaces in a cod-processing facility. At the end-of-day rinse, mean TMC reductions were also greater with ozonated water, but the difference was not statistically significant after Holm’s correction. The absence of a significant advantage during the lunch rinsing and the subsequent increases in microbial counts support assessing immediate rinsing performance separately from microbial accumulation during resumed production. Ozonated water may therefore serve as a complementary hygiene intervention during production breaks, alongside conventional end-of-day cleaning and disinfection. Any potential reduction in chemical disinfectant consumption would require specific evaluation.

## Figures and Tables

**Table 1:** Daily cleaning and sanitation schedule applied to the Target Batcher portioning machine under standard operating conditions.

Time	Cleaning Operation
08:30	Start of production
10:30	Mid-morning break—high-pressure cold-water rinse
13:00	Lunch break—high-pressure cold-water rinse
17:30	End of production—high-pressure cold-water rinse followed by sanitation with a chlorine-based foaming agent and final water rinse

**Table 2:** Mean (± SD) Total Mesophilic Counts (TMC) and Total Psychrotrophic Counts (TPC) expressed as log CFU/cm^2^ at the different sampling points throughout the working day under tap water and ozonated water cleaning procedures.

Sampling Point	Water TMC	Ozone TMC	Water TPC	Ozone TPC
**Start of production**	1.32 ± 1.54	1.89 ± 1.36	1.35 ± 1.58	1.62 ± 1.77
**Pre-morning**	2.87 ± 0.80	2.88 ± 0.80	3.28 ± 0.85	3.05 ± 0.59
**Post-morning**	2.11 ± 0.54	0.95 ± 1.43	2.62 ± 0.83	0.88 ±1.33
**Pre-lunch**	2.85 ± 0.73	2.40 ± 0.76	3.49 ± 1.16	2.38 ± 1.07
**Post-lunch**	1.84 ± 0.47	0.86 ±1.20	2.12 ± 0.86	0.26 ±1.41
**Pre-end**	2.69 ± 0.98	2.86 ± 0.91	3.44 ± 0.84	3.21 ± 0.69
**Post-end**	1.49 ± 1.20	0.65 ± 1.47	2.69 ± 0.65	1.08 ± 1.05
**End after chlorine**	1.80 ± 0.81	2.37 ± 0.42	2.17 ± 0.82	1.76 ±1.17

## Data Availability

The data supporting the findings of this study are available from the corresponding author upon reasonable request.

## Abbreviations

The following abbreviations are used in this manuscript:

TMC	Total mesophilic counts
TPC	Total psychrotrophic counts
MRD	Maximum recovery diluent
PCA	Plate count agar
CFU	Colony-forming units
SD	Standard deviation
RM-ANOVA	Repeated measures analysis of variance
